# Fracture resistance and 3D finite element analysis of machined ceramic crowns bonded to endodontically treated molars with two planes versus flat occlusal preparation designs: an *in vitro* study

**DOI:** 10.12688/f1000research.19455.1

**Published:** 2019-07-08

**Authors:** Omnia Nabil, Carl Hany Halim, Ashraf Hassan Mokhtar

**Affiliations:** 1 Department of Fixed Prosthodontics, Faculty of Dentistry, Cairo University, Cairo, 11553, Egypt

**Keywords:** Two planes occlusal preparation, Flat occlusal preparation, Endodontically treated molars, Ceramic-crown tooth structure, Fracture resistance, 3D Finite Element Analysis.

## Abstract

**Background: **The flat occlusal preparation design (FOD) of posterior teeth offers promising results of fracture resistance and stress distribution, but its application in vital teeth is limited as there may be a danger of pulp injury. Although this danger is omitted in endodontically treated teeth, there is no research work assessing the impact of FOD on the fracture resistance and distribution of stresses among these teeth. The aim of this study was to assess the impact of FOD of endodontically treated molars on the fracture resistance and distribution of stresses among a ceramic crown-molar structure when compared to the two planes occlusal preparation design (TOD).

**Methods:** 20 human mandibular molars were endodontically treated and distributed equally to two groups: Group I (TOD) and Group II (FOD). Ceramic CAD/CAM milled lithium disilicate (IPS e.max CAD) crowns were produced for all preparations and adhered using self-adhesive resin cement. Using a universal testing machine, the fracture resistance test was performed. The fractured samples were examined using a stereomicroscope and scanning electron microscope to determine modes of failure. Stress distribution was evaluated by 3D finite element analysis, which was performed on digital models of endodontically treated mandibular molars (one model for each design).

**Results:** Group II recorded statistically non-significant higher fracture resistance mean values (3107.2± 604.9 N) than Group I mean values (2962.6 ±524.27 N) as indicated by Student’s t-test (t=0.55, p= 0.57). Also, Group II resulted in more favorable failure mode as compared to Group I. Both preparation designs yielded low von-Mises stresses within the factor of safety. However, the stress distribution among different layers of the model differed.

**Conclusions: **FOD having comparable fracture strength to TOD and a more favorable fracture behavior can be used for the preparation of endodontically treated molars.

## Introduction

Endodontic treatment weakens posterior teeth and ought to be covered by crowns ^1^. The capability of these crowns to bear load relies on the preparation of an appropriate design and the selection of a crown material with adequate fracture strength and thickness ^2^.

In vital teeth, the anatomic occlusal preparation design is followed such that the occlusal surface is reduced uniformly, maintaining the cusps, fissures and normal inclined planes but at a reduced height. This aids in minimizing the risk of pulp injury. In contrast, in non-vital teeth, this design can be modified such that the occlusal surface is prepared in two planes (buccal and lingual planes) ^3^.

A flat prepared occlusal surface provides less quantitative and better qualitative stresses when compared to an anatomically prepared surface ^4^. Also, an anatomically prepared occlusal surface follows old preparation configurations for non-bonded crowns and more concern needs to been given to the functioning of bonded crowns, which can preserve tooth structure ^5^.

The aim of our research was to assess the impact of a flat prepared occlusal surface (FOD) of endodontically treated molars on the fracture resistance and the distribution of stresses among the ceramic crown-molar structure when compared to a two planes prepared occlusal surface (TOD).

The hypothesis of our research was there would not be significant differences in both fracture resistance and developed stresses of the ceramic crown-molar structure of FOD when compared to TOD of endodontically treated molars (null hypothesis).

## Methods

### Ethical approval

This study was approved by the Research Ethics Committee of the Faculty of Dentistry, Cairo University. Approval number: 15636 ( *Extended data*).

Extracted teeth were obtained from the outpatient clinic, Oral Surgery Department, Faculty of Dentistry, Cairo University. Any researcher in the institute can obtain extracted teeth that meet the criteria of the research without requiring the researcher to contact the patients, since the patients give their consent for their extracted teeth to be used in future research when they are extracted.

### Sample size calculation

Student’s t-test was performed to compare two groups (Group I: TOD; Group II: FOD), as per a previous study by Shahrbaf *et al*. ^5^. The primary outcome of this study is the fracture resistance with an estimated mean value of 407.7±82.7 N for the control group (Group I) and 661.1±190 N for the test group (Group II) (effect size =1.7 with alpha 0.05 and power =0.8). Priori power showed that the required sample size should be above 14 (7 in each group) (calculated using G*power release 3.1.9.2). Accordingly, a total sample size of 20 (10 per group) was performed.

### Sample fabrication

***Teeth collection, endodontic treatment and coronal build up*.** In total, 20 human mandibular molars free of caries, defects and cracks were chosen. The mean measurement between the maximum convexity on the buccal and lingual surfaces of the selected teeth differed by ≤ 2.5% (as measured using a digital caliber (Harbor Freight Tools, CA, USA)) ^6^. The teeth were kept in distilled water after ultrasonic scaling which was done to remove any remnants. Conventional access cavities were prepared in all teeth. Manual preparation and enlargement of root canals was performed until size # 25 (MANI, Japan) ^7, 8^. Rotary root canal preparations were then performed with a series of ProTaper Ni-Ti rotary instruments (Dentsply Maillefer, Switzerland). The matched gutta percha points (Dentsply Maillefer, Switzerland) and resin based sealer (Adseal; META BIOMED Co, Korea) were used for obturation and the excess gutta percha was removed by a heated plugger ^9^. Coronal cavities were then treated with 37% phosphoric acid Etch (Spident, USA) for 15 seconds, rinsed for 10 seconds and dried gently using a cotton pellet. A layer of light cure bonding agent (Adper Single Bond Plus Adhesive; 3M ESPE AG, Germany) was applied with gentle agitation and light cured for 10 seconds, then packable composite resin (3M Filtek Z250 XT Nano hybrid composite resin; 3M Deutschland GmbH, Germany) was incrementally added and light cured ^10^.

***Teeth mounting, grouping and preparation*.** A plastic ring (2.5 cm in diameter and 2 cm in length) was utilized to mount the teeth in epoxy resin (CMB, Egypt) and a custom made paralleling device ( *Extended data*) was used to allow accurate vertical centralization of the tooth in the ring. The mounted teeth were randomly distributed into two equal groups as follows:

Group I (TOD): Prepared teeth with two planes occlusal surface.

Group II (FOD): Prepared teeth with flat occlusal surface.

**Figure 1.**
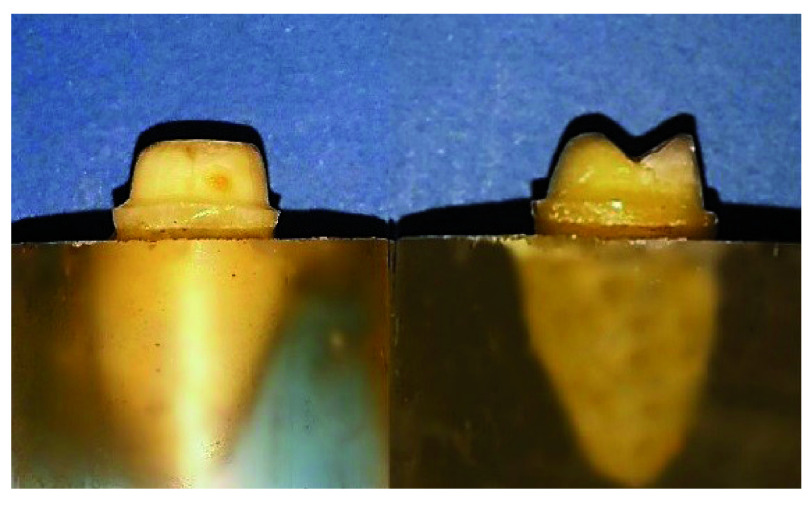
Flat occlusal preparation design (left) and two planes occlusal preparation design (right).

A special milling machine (AF30 Nouvag, Switzerland) was used to prepare all the teeth by the same operator ( Figure 1). The preparation parameters are detailed in Table 1.

**Table 1.** Parameters of teeth preparation.

	Group I (TOD)	Group II (FOD)
Axial	1.5 mm	1.5 mm
Finish line	1 mm rounded shoulder	1 mm rounded shoulder
Taper	6°	6°
Occlusal surface	Two planes occlusal reduction (1.5 mm from occlusal center)	Flat occlusal reduction (1.5 mm from occlusal center)

***Crown fabrication and cementation*.** A CAD/CAM system (CEREC AC; Sirona, Germany) was used for the fabrication of all crowns. Each prepared tooth was scanned using the CEREC Omnicam and design was carried out using CEREC Premium 4.4 software. The distance between central groove of restoration and the occlusal surface of tooth was standardized at 1.5 mm in all restorations. Milling of the crowns was done from Lithium disilicate blocks (IPS e.max CAD; Ivoclar Vivadent AG, Principality of Liechtenstein) in 4-axis milling machine CEREC MC XL. Finally, crowns were fully crystallized and glazed in Programat P510 furnace (Ivoclar Vivadent AG, Principality of Liechtenstein).

Surface treatment of the fitting surface of each crown was done as follows: Porcelain Etch (BISCO, USA) application for 20 seconds, followed by water rinsing and drying, then Silane (BISCO, USA) application for 60 seconds followed by air drying for 5 seconds. Self-adhesive resin cement (RelyX Unicem; 3M ESPE AG, Germany) was used for cementation.

Each crown was seated on its corresponding tooth and held with light pressure. The excess cement was cleared by an explorer after 2 seconds of tack-curing, then glycerine based gel (K-Y Jelly; Johnson & Johnson, USA) was applied at the margins of crown to prevent the oxygen inhibiting layer. A 5 kg load was applied parallel to the long axis of each tooth during cementation using a custom made loading device ( *Extended data*) ^11, 12^. The load was applied for 5 minutes to allow the cement to self-cure as recommended by the manufacturer. This was followed by final curing of axial and occlusal surfaces with light cure for 20 seconds.

### Fracture resistance test

The test was carried out using a computer-controlled material testing machine (Instron, Model 3345; Instron industrial, USA). Each sample was tightened to the lower fixed compartment by screws. A compressive load was applied on the occlusal surface utilizing a metallic rod with round tip (5.8 mm diameter) attached to the upper movable compartment traveling at cross-head speed of 1mm/min with tin foil sheet in-between to achieve homogenous stress distribution and to minimize the transmission of local force peaks. The load to fracture was recorded in Newtons (N) using Instron Bluehill Lite Computer Software version 2 (Instron, USA). The fracture was manifested by an audible crack and confirmed by a sharp drop at load-deflection curve.

### Statistical analysis

Data from the two groups were gathered, arranged and analyzed using SPSS (version 21; IBM, USA).

### Microscopic examination of fractured samples

High-performance Leica MZ6 Stereomicroscope (Meyer Instruments, USA) with 6.3:1 zoom was used to evaluate the fracture mode of the samples, indicating areas of interest for further examination under a scanning electron microscope (SEM; Model Quanta 250 Field Emission Gun; FEI Company, The Netherlands) attached with Energy Dispersive X-ray Analyses unit, with 30 KV accelerating voltage, 70X, 250X magnification and resolution of 1nm.

### 3D modeling and finite element analysis

***3D designing of models***

**3D scanning.** Two of the prepared samples (one for each group) and their corresponding crowns were scanned before cementation to produce models with real geometrical measures. 3D reconstruction from cone beam computed tomography (CBCT) data of the teeth scans was found had high linear, volumetric, and geometric accuracy ^13^. A CBCT scanner (Next Generation iCAT scanner; ISI, USA) was used to obtain CBCT images in this research. After scanning, data were exported in DICOM format. Mimics software version 17 (Materialise, Belgium) was used ^a^ for segmenting the scanned objects into separate elements. A definitive threshold level was set to most clearly show each element of the scanned samples with minimal interference from the surrounding structures, and once segmentation was completed the software automatically calculated the element’s volume. The resulting STL files were opened separately on Meshmixer software version 3.3.15 (Autodesk Inc., USA) for the improvement of mesh quality and its refinement.

**Figure 2.**
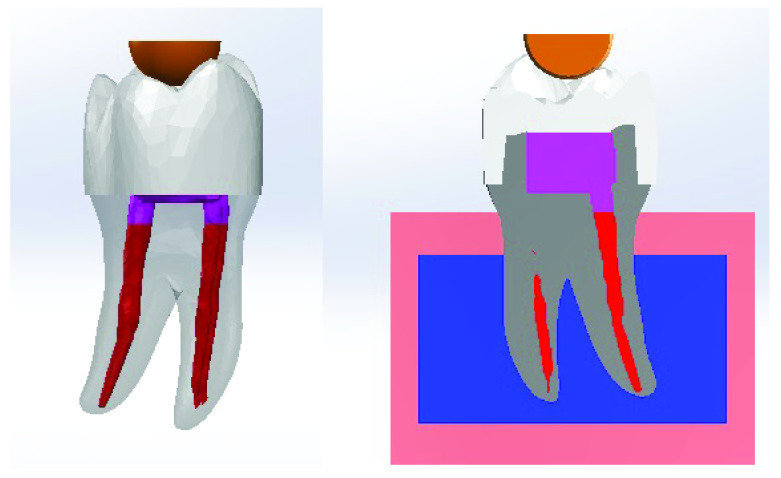
3D CAD model (left). The model embedded in bone (right).

**Reverse engineering and assembly.** Reverse engineering was performed by NX software version 10 (Siemens PLM, Texas, USA) ^b^. The refined STL files were imported into the software and converted into solid parts. Then, cortical bone and cancellous bone were drawn as solid parts in cylindrical shapes followed by their superimposition together for Boolean subtraction. Finally, all the produced solid parts were superimposed together for Boolean subtraction and periodontal ligaments were modeled with 0.2 mm thickness to allow a fully defined simulation methodology. The generated 3D CAD geometry of all parts were then assembled using Solidworks software 2017 (Dassault Systèmes SolidWorks Corporation, France) ^c^ to produce the 3D CAD models, one for each group ( Figure 2).

***Finite element analysis*.** Finite element analysis (FEA) was carried out by ANSYS R16.2 software (ANSYS, Canonsburg, USA) ^d^ using the 3D CAD models. It included 3 phases:

**Pre-processing phase.** The type of element was defined as Solid 10 node 187. All the materials’ properties were set as isotropic, homogenous and linear elastic. The modulus of elasticity and Poisson’s ratio of each material were gathered and were uploaded to the software ( Table 2). A perfect rigid bonding with no-slip condition between all the elements was simulated. Each model was divided into small parts called elements connected together at points called nodes forming a mesh structure. Parabolic tetrahedral solid elements were used to form a fine solid mesh. The overall number of elements and nodes was recorded ( Table 3).

**Table 2.** Physical properties of each component of the model.

Material	Modulus of elasticity (GPa)	Poisson’s ratio
**Dentin**	18.6 ^14^	0.31 ^14^
**Periodontal ligaments**	0.0000689 ^15^	0.45 ^15^
**Cancellous bone**	1.37 ^14^	0.30 ^14^
**Cortical bone**	13.7 ^14^	0.30 ^14^
**IPS e.max CAD crown**	96 ^16^	0.23 ^16^
**Filtek Z250 composite**	14 ^17^	0.31 ^17^
**Gutta percha**	0.14 ^15^	0.45 ^15^
**Rely X Unicem** **cement**	4.9 ^16^	0.30 ^16^

**Table 3.** The overall number of elements and nodes.

	Element	Node
**Group I model**	493788	706944
**Group II model**	439230	630975

**Processing phase.** Following the creation of the 3D meshes, a zero displacement boundary condition was set at all nodes of the cortical bone that were confined in X, Y, and Z directions. Posterior fixed restorations ought to have the capability to tolerate a 500 N occlusal load ^18, 19^. Accordingly a load of 500 N was applied by a ball model of diameter 5.8 mm equal to that of metallic rod ball of the universal testing machine used for fracture resistance test in this study. The occlusal surface of the crown was loaded at the inner inclines of the mesiobuccal, distobuccal and mesiolingual cusps ^20^. The load was applied in vertical direction as the masticatory forces directed onto molar teeth is mostly vertical, while through the anterior guidance and lateral guidance, they are guarded by the anterior teeth ^21^.

**Post-processing phase.** The output of the processing phase was displayed as graphical output and numeric output.

## Results

### Fracture resistance

Fracture resistance results as a function of preparation design are summarized in Table 4.

**Table 4.** Descriptive statistics of fracture resistance results as a function of preparation design.

Variables		Group I	Group II
Mean		2962.6	3107.2
95% Confidence Interval for Mean	Lower Bound	2587.6	2674.5
	Upper Bound	3337.7	3539.9
Std. Deviation		524.2	604.94
Minimum		2206.01	2253.5
Maximum		4014.7	4153.4

It was found that the fracture resistance mean ± SD value recorded for Group I was 2962.6 ± 524.2 N, with minimum value of 2206.01 N and maximum value of 4014.7 N. For Group II, the mean ± SD value recorded was 3107.2 ± 604.94 N, with minimum value of 2253.5 N and maximum value of 4153.4 N.

**Figure 3.**
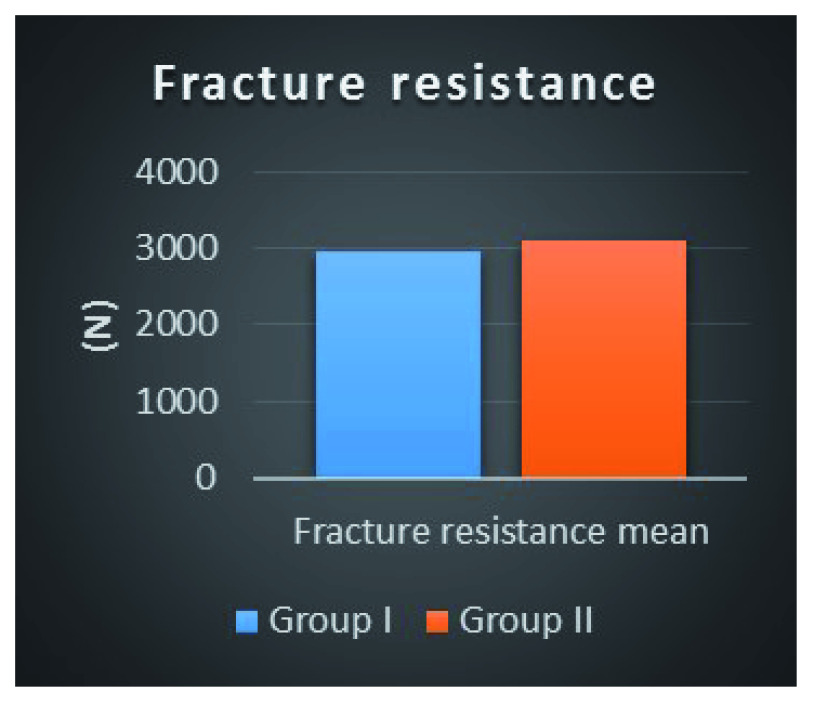
Column bar chart of fracture resistance mean values for both preparation designs.

The difference between the mean fracture resistance between the two groups was non-statistically significant as indicated by Student’s t-test (p=0.57) ( Figure 3).

### Fractographic analysis of fractured samples

**Figure 4.**
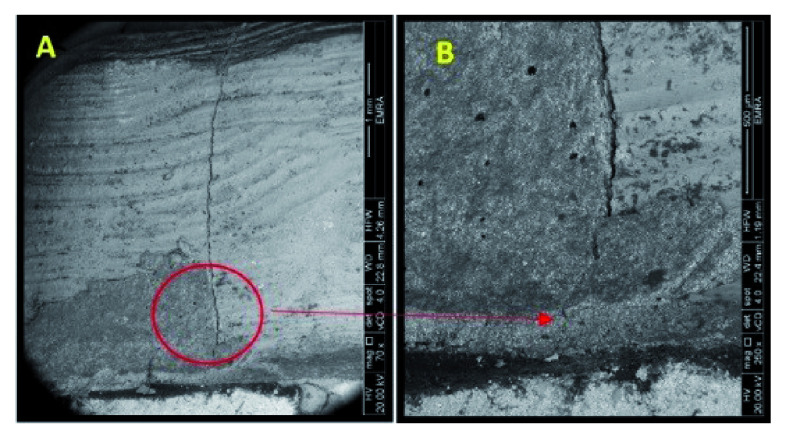
SEM image revealed the crack extension through the finish line (A: 70X, B: 250X).

The behavior of the samples upon fracture differed ( Table 5). Five different modes were observed and categorized as restorable or non-restorable according to their relation to the cemento-enamel junction (CEJ). A fracture that ends before CEJ, implying that even after the occurrence of fracture, the tooth can be saved is a restorable one, while fractures that are non-restorable extend beyond CEJ and extraction of the tooth is expected ^22^. The extensions of the cracks were verified by SEM ( Figure 4). The results are tabulated in Table 6 and classified as restorable and non-restorable.

**Table 5.** Classification of the modes of failure. Restorable remaining tooth structure: modes I, II and IV; non-restorable remaining tooth structure: modes III and V.

Mode of failure	I	II	III	IV	V
**Descriptive form**	Crack limited to occlusal half.	Crack extended to cervical half.	Crack extended through and beyond cervical finish line.	Fractured segment of coronal part of tooth.	Fractured segment of coronal part and root of the tooth.
**Descriptive** **Stereomicroscopic** **photo**	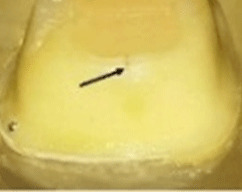	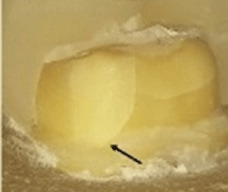	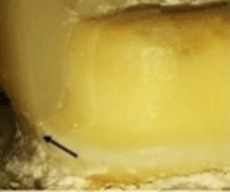	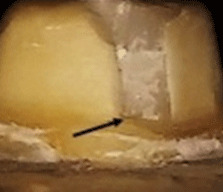	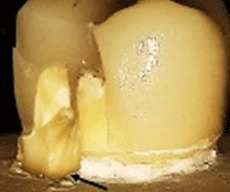

**Table 6.** Number of samples of Groups I and II relative to mode of failure, with restorability reported.

Behavior		Group I (N)	Group II (N)
Mode of failure	I	0	3
	II	2	2
	III	3	3
	IV	0	1
	V	5	1
Restorability	Restorable	2	6
	Non-restorable	8	4

### 3D FEA

In each model, FEA revealed stresses at every node. These results were presented as stress contours overlaid on the model. The numeric data of stress, deformation and safety factor in the models were calculated and transformed into color graphics.

***Equivalent (von-Mises) stress*.** The "von-Mises Stress" at different areas were calculated and compared ( Table 7). The stress distribution values were generally found to be low.

**Table 7.** The von-Mises stress values for each model. Red dot marks the higher value.

			Group I (Two planes) MPa	Group II (Flat) MPa
Area				
Tooth (Dentin)	Occlusal Surface	Mesiobuccal cusp	4.10	6.36 •
		Mesiolingual cusp	5.15	6.01 •
		Mesial marginal ridge	4.57	8.66 •
		Distal marginal ridge	4.63 •	3.15
		Distobuccal cusp	4.27 •	3.96
		Distolingual cusp	4.30 •	3.03
		Distal cusp	3.92 •	2.32
	Axial walls	Mid buccal	0.26	1.86 •
		Mid distal	1.76	2.64 •
		Mid lingual	3.07	3.37 •
		Mid mesial	3.46 •	2.40
	Finish line	Mid buccal	4.66 •	3.64
		Mid distal	1.86	2.72 •
		Mid lingual	5.66 •	4.36
		Mid mesial	6.24 •	3.17
	Root (neck of the tooth)	Buccal	3.00	4.14 •
		Lingual	6.23	6.62 •
		Distal	2.36	2.57 •
		Mesial	5.87 •	4.76
		Furcation	5.37 •	5.24
Crown center			22.60	36.50 •
Core center			22.60	36.50 •
Gutta percha		Mesiobuccal	0.14640 •	0.13680
		Mesiolingual	0.00736 •	0.00735
		Distal	0.00653 •	0.00625

**Figure 5.**
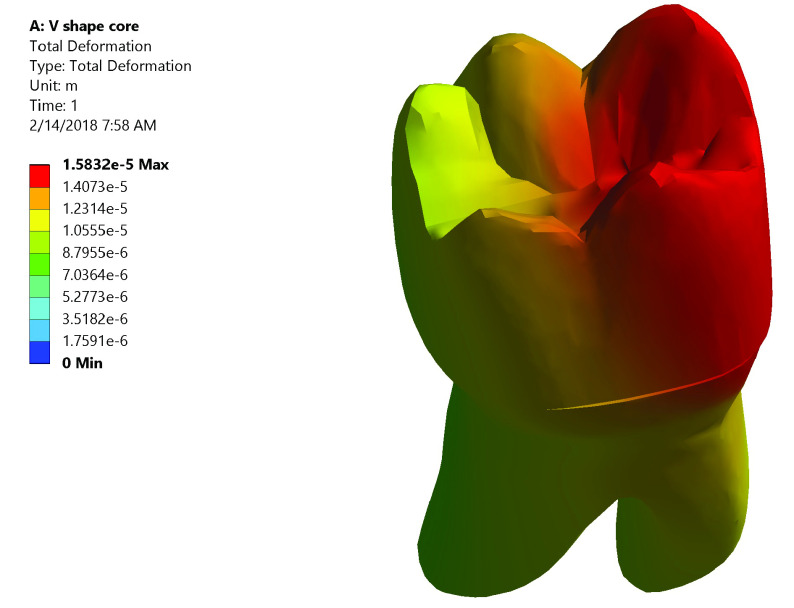
Color graphics showing total deformation of Group I.

**Figure 6.**
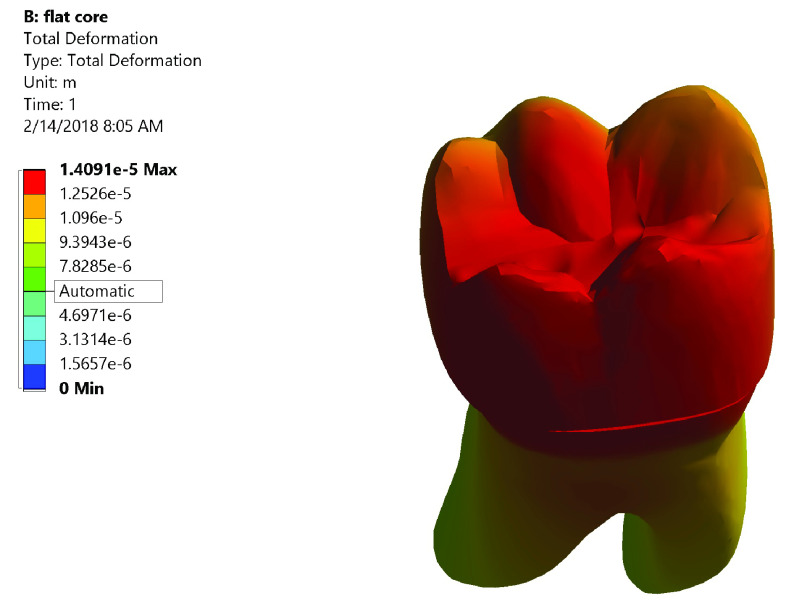
Color graphics showing total deformation of Group II.

***Total deformation*.** The maximum value of total deformation denoted by the red color in Group I was 0.0158 mm. It was concentrated on the mesial half of the coronal portion of model (Crown, dentin and core) and the root (neck of the tooth) at the mesial surface and the mesial half of the lingual surface ( Figure 5). While in Group II the maximum value of total deformation was 0.01409 mm. It was located on middle and the lingual half of coronal portion of model and the lingual neck of the tooth with mesiolingual and distolingual line angles ( Figure 6). When comparing both groups, Group II yielded less total deformation values than Group I.

**Figure 7.**
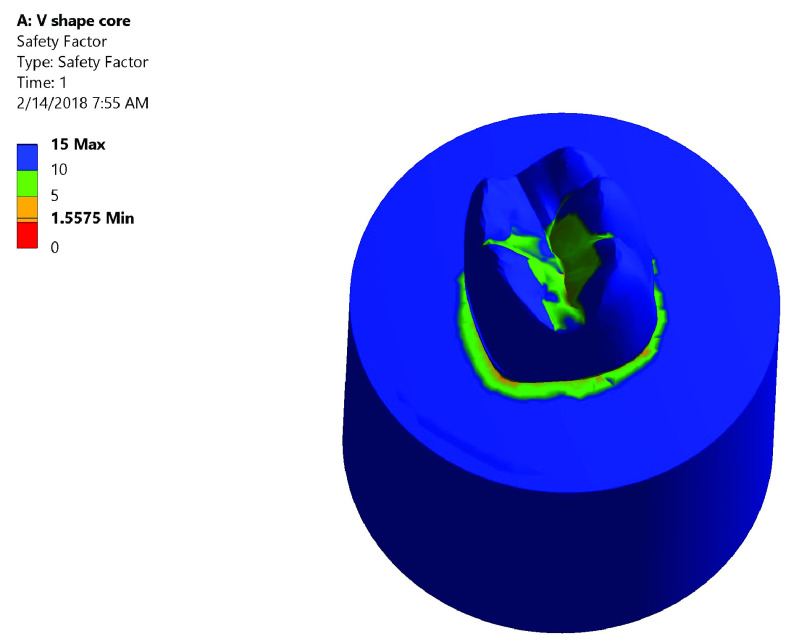
Color graphics showing safety factor of Group I.

**Figure 8.**
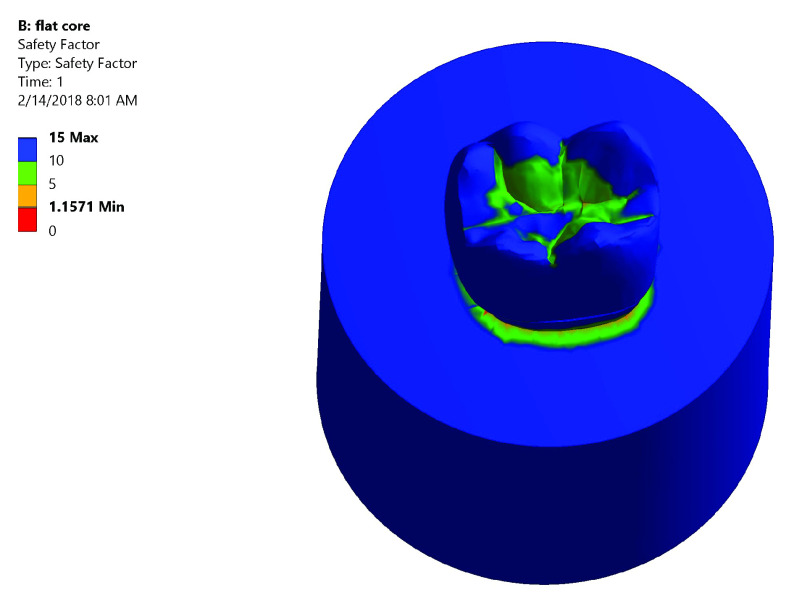
Color graphics showing safety factor of Group II.

***Safety factor*.** Both groups had high safety factor where the maximum equivalent stress was less than the stress limit. In Group I: the lowest safety factor recorded was 1.5575 was at the mesial neck of the tooth denoted by the orange color ( Figure 7). In Group II: the lowest safety factor recorded was 1.1571 was at the lingual neck of the tooth denoted by the orange color ( Figure 8).

### Correlation between fracture behavior and stress distribution

**Figure 9.**
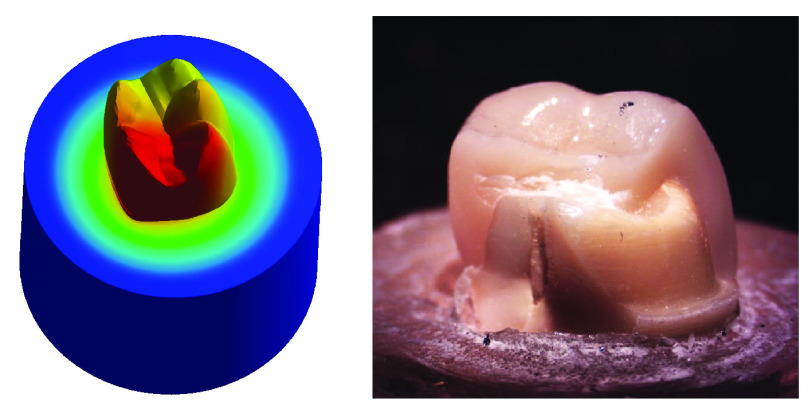
Correlation between total deformation (left) and fracture behavior (right) of Group I.

The stress distribution among different layers of the model differed as well as areas of total deformation. This was correlated with the fracture behavior of samples of both groups as follows:

***Group I ( Figure 9)*.** The stress values were high at mid-mesial and mid-lingual axial walls, mid-mesial and mid-lingual finish line, and root’s mesial surface. Maximum total deformation was concentrated in the mesial half of the coronal portion of model (crown, dentin and core) and the neck of the tooth at the mesial surface and mesial half of the lingual surface.

Upon observation of fracture behavior of Group I samples of fracture resistance, the failure in most of the samples occurred at the mesial half of the crown-tooth structure including the finish line and the root (neck of the tooth).

**Figure 10.**
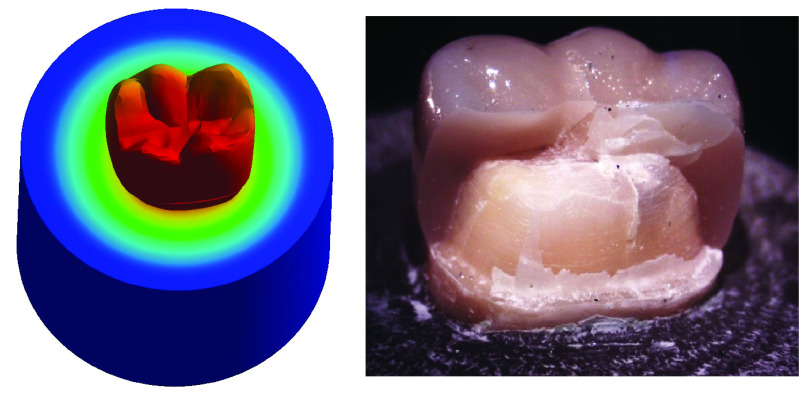
Correlation between total deformation (left) and fracture behavior (right) of Group II.

***Group II ( Figure 10)*.** The centers of the crown and the underlying core material generated high stress values upon load application. Also, at mesiobuccal cusp, mesiolingual cusp, mesial marginal ridge, and root’s lingual surface stresses were high. Maximum total deformation was located at the middle and the lingual half of coronal portion of model (crown, dentin, and core) and the lingual neck of the tooth with mesiolingual and distolingual line angles.

Upon observation of fracture behavior of Group II samples of fracture resistance, the failure in more than half of the samples occurred at the lingual half of the crown-tooth structure, not including the finish line and the root (neck of the tooth).

## Discussion

Previous research has generally given minimal concern to the impact of the preparation configuration on the ability of the crown-tooth structure to resist fracture and distribute stresses, and instead has mainly targeted the crown material itself ^5^. Thus, the focus of this study was to specifically address the impact of the prepared occlusal surface configuration comparing two planes occlusal preparation (TOD) versus flat occlusal preparation (FOD) of endodontically treated molars.

In our study, mandibular molars were chosen due to their high incidence for developing caries, their subjection to strong occlusal loads and greater susceptibility to fracture ^23^. Single-cone obturation technique was followed to exclude the inordinate cutting of dentin needed to ease the entry of the endodontic plugger in vertical warm condensation technique and the wedge-acting stresses of the spreaders during lateral cold condensation technique ^24^. Lithium disilicate ceramic was chosen for crown fabrication. This ceramic material features appropriate mechanical and esthetic qualities for making monolithic restorations, allowing conservative tooth preparation and simplicity of production ^25^. Self-adhesive resin cement was selected as it was revealed that the use of adhesive cement raised the fracture resistance by almost 26% when utilized with lithium disilicate glass ceramic as compared to non-adhesive cement ^26^.

Periodontal ligament (PDL) was not simulated. Upon the application of a static load, no difference was anticipated in the resistance of teeth to fracture whether their roots were covered with a PDL simulating material or not. Moreover, the thickness of the silicone PDL simulating material used to cover the roots was found to be thicker than the normal PDL thickness. Also, with the difficulty to unify the thickness of this material, there was no control on the movability of the investigated teeth and more drawbacks were expected ^27^.

Investigation of the fracture resistance of crown-molar structure was the primary study objective. Static load fracture test was employed in an occluso-axial direction, which is viewed as the most well-known strategy for testing the integrity of a structure ^5^.

Checking the areas of stress is crucial as stress, regardless of if it is beneath the point of failure, is considered as a noteworthy reason for propagation of a crack and henceforth failure. 3D FEA has been used to investigate stress bearing and handling capabilities of various restoration materials and shapes in a safe and time saving method ^18^.

FEA simulated the test of fracture resistance performed in this investigation to demonstrate the generated points of stress subsequent to load application. A good-bond interlayer condition was assumed between the distinctive layers in the model. 

Our results failed to reject the null hypothesis that FOD of endodontically treated molars would not differ in both fracture resistance and the generated stresses of the ceramic crown-molar structure when compared to TOD.

The results of fracture resistance test displayed no significant difference statistically between both tested groups with an average ˃ 2900 N, which surpasses the average and maximum biting force reported in the mouth (100-600 N) ^28^.

Also, these results exceeded those of other studies. According to Nordahl *et al.* ^29^ the mean fracture resistance of e.max lithium disilicate crowns of posterior molars of thickness 1.5 mm was 1,431 N while according to Yu *et al*. ^22^ it was 1827.3 N. This can be attributed to the exposure of crowns in both studies to artificial aging before loading until fracture. The exposure of the crowns to aging was stated in various investigations to diminish the resultant fracture loads significantly ^29, 30^.

Regardless of the close and clinically satisfactory fracture resistance results of both investigated designs, the failure mode varied. It was found that 20% of Group I (TOD) had undergone restorable fractures, while in Group II (FOD) this was 60%. This denotes that FOD showed better stress distribution, thus more favorable fracture behavior.

The 3D FEA data recorded in this study showed that both preparation designs yielded low von-Mises stresses within the factor of safety of the model. In both groups, high stresses and deformation were induced in the lingual surface of molars. This was justified by the weakening effect of the hard tissue loss on the mandibular molars, which might predispose the lingual wall to fracture due to unfavorable distribution of stresses during chewing ^31^. This was confirmed by Oyar *et al.* ^2^ who found high stress values were generated in the lingual dentin regions in both anatomic and non-anatomic occlusal preparation designs.

Upon observation of fracture behavior of samples of fracture resistance test and correlating it with the results of the FEA, the following were concluded: Group I (TOD) developed lower stresses in the center of the crown and the core, and the stresses increased upon moving apically towards the root with greater influence on the mesial half of the model. This explained the failure pattern in most of the samples that occurred at the mesial portion of the crown-molar structure including the finish line and the root (neck of the tooth). While Group II (FOD) developed higher stresses in the center of the crown and the core, and the stresses decreased upon moving apically towards the root with greater influence on the lingual half of the model. This explained the failure pattern in more than half of the samples which occurred at the lingual portion of the crown-molar structure without the inclusion of finish line and root (neck of the tooth).

Our results differed than other studies regarding the stress distribution pattern. According to, Oyar *et al*. ^32^ the anatomically prepared design yielded better stress distribution in dentin while the non-anatomic design yielded better distribution and less amount of stresses in the porcelain structure. Their study was carried out on mandibular second premolars restored by metal-ceramic crowns.

While Shahrbaf *et al*. ^4^ concluded that the flat occlusal configuration presented lower stresses than the anatomic configuration in all layers and a more favorable distribution. Their study was carried out on maxillary first premolars with variable amount of occlusal reduction, such that 2 mm even reduction for the anatomic design and 1.2 mm reduction from the central groove for the flat one.

Oyar *et al*. ^2^ found that different designs did not result in differences in the generated stresses in tooth (dentin, pulp) and bone. However, the anatomic design crown had the highest value of stresses and this was attributed to the crown thickness, which was less than that of the non-anatomic. Their study was conducted on mandibular second premolar teeth.

In the current researchers’ opinion, the comparable results obtained among the two tested groups in the present study could be attributed to multiple factors: First, unifying the ceramic thickness at the fissure depth to 1.5 mm thickness, which is the most critical area for failure; second, selecting lithium disilicate monolithic crowns as the material of choice with its well-known high mechanical properties in terms of flexural strength and good bonding potential to tooth and composite resin; third, conducting adhesive bonding protocol using adhesive composite resin cement, which has a positive impact on overall fracture resistance of the ceramic-molar structure.

The fracture resistance test used in this research is considered a limitation, as it does not precisely mimic the load application inside patient’s mouth, which is a cyclic loading process. Also, another limitation is the consideration of having a perfect bond between the crown and tooth in FEA method.

## Conclusions

1. Flat and two planes occlusal preparation designs of endodontically treated molars had fracture resistance values surpassing the average and maximum biting force reported in the mouth.

2. Flat and two planes occlusal preparation designs of endodontically treated molars showed stress values within the safety factor when subjecting the models to the average biting force.

3. Flat occlusal preparation design showed more favorable mode of failure as compared to two planes occlusal preparation design based on the fractographic and 3D finite element analyses.

4. Flat occlusal preparation design can be used safely with endodontically treated molars.

### Clinical significance

The occlusal reduction of endodontically treated molars can influence the functioning of the crown-molar structure. The occlusal surface preparation design has to strengthen the prepared tooth to sustain the forces being subjected to and, upon failure, it favors a restorable mode.

This study revealed that restorable fractures were higher in flat occlusal preparation design than two planes occlusal preparation design. Therefore, clinicians may choose the flat occlusal preparation design to improve the clinical performance and longevity of the restored endodontically treated molars.

### Recommendations

1. Clinical studies comparing the behavior of the two tested designs.

2. Designing the same research using other contemporary metal free crown materials (hybrid materials, polycrystalline materials), which may yield different outcomes.

## Data availability

### Underlying data

Open Science Framework: Fracture Resistance and 3D Finite Element Analysis of Machined Ceramic Crowns Bonded to Endodontically Treated Molars with Two Planes versus Flat Occlusal Preparation Designs. “ *In-Vitro* Study”, https://doi.org/10.17605/OSF.IO/WMRU4 ^33^.

This project contains the following underlying data:

- 3D FEA results (.xlsx files)
- Fracture resistance data (.xlsx file)
- SEM raw data (.doc file)
- FEA output (.mechdat files)
- Solidworks software 2017 files, 3D models (.x_t files)
- Instron Lab fracture resistance output report

### Extended data

Open Science Framework: Fracture Resistance and 3D Finite Element Analysis of Machined Ceramic Crowns Bonded to Endodontically Treated Molars with Two Planes versus Flat Occlusal Preparation Designs. “ *In-Vitro* Study”, https://doi.org/10.17605/OSF.IO/WMRU4 ^33^.

This project contains the following extended data:

- Ethical approval document
- Custom made paralleling device
- Custom made loading device

## Footnotes

^a^ Free alternative software that could be used for this analysis is ITK-SNAP.

^b^ Free alternative software that could be used for this analysis is FreeCAD.

^c^ Free alternative software that could be used for this analysis is FreeCAD.

^d^ Free alternative software that could be used for this analysis is OpenFoam V6.
