# Supplementary material for: Fracture resistance and 3D finite element analysis of machined ceramic crowns bonded to endodontically treated molars with two planes versus flat occlusal preparation designs: an in vitro study
Source: F1000Res. 2021 Jun 11;8:1020. Originally published 2019 Jul 8. [Version 2] doi: 10.12688/f1000research.19455.2 (PMC9706146; doi:10.12688/f1000research.19455.2)
Supplement: Peer review history

# Peer review: Reviewer response for version 2

**DOI:** 10.5256/f1000research.56912.r156322

**Published:** 2022-11-28

**Authors:** Mostafa Aboushahba

**Affiliations:** 1 Fixed Prosthodontics Faculty of Oral and Dental Medicine, Future University in Egypt, Cairo, Egypt

I am very pleased with the authors' work and the scientific content of the article. It is of great clinical value and gives preliminary answers to a long going question as to whether to prepare a flat occlusal surface or anatomical occ surface.

 For the discussion:

- Can you add when to post or not to post; justify why didn't you use a post in your methodology.
- Can you also add that the flat occlusal surface had better results yet non-significant, that may be due to the structural durability of the material since the flat occlusal surface provides more restoration thickness than the anatomical.

Recommendations:

- Test cyclic loading as it resembles the oral environment more.
- Test the retention as I expect due to the reduction of occlusion-gingival height of the prep it might reduce the resistance and retention.

Is the work clearly and accurately presented and does it cite the current literature?

Yes

If applicable, is the statistical analysis and its interpretation appropriate?

Yes

Are all the source data underlying the results available to ensure full reproducibility?

Yes

Is the study design appropriate and is the work technically sound?

Yes

Are the conclusions drawn adequately supported by the results?

Yes

Are sufficient details of methods and analysis provided to allow replication by others?

Yes

Reviewer Expertise:

Associate Professor Fixed Prosthodontics  Future University in Egypt FUE

I confirm that I have read this submission and believe that I have an appropriate level of expertise to confirm that it is of an acceptable scientific standard.

# Peer review: Reviewer response for version 1

**DOI:** 10.5256/f1000research.21329.r50923

**Published:** 2020-03-09

**Authors:** Tarek Salah

**Affiliations:** 1 Fixed Prosthodontics Department, Faculty Of Dentistry, Ain Shams University, Cairo, Egypt

This is a very interesting research and of high clinical interest and I really appreciate the work of the authors. It is definitely publishable material after some revisions. 

**Materials and Methods:**

- Why didn’t you consider doing mechanical cyclic loading before the fracture resistance testing? I think it would have been more clinically significant.
- More details are needed concerning teeth dimensions.
- Access cavity design and dimensions should be also described as all these details are important in behavior of the teeth during fracture resistance testing. And also, please mention the master apical file size.
- To which level were the teeth mounted in the epoxy resin?
- There should be more elaboration on specifics of teeth preparation designs, especially occlusal designs. A diagram would be highly beneficial. The photograph is not that descriptive.
- What were the details of crowns dimensions and standardization during designing?

 

**Testing:**

- Indicate the points of load application during fracture resistance testing?

 

**Statistical analysis:**

- There are no details. What type of tests or analysis did you perform?

**FEA:**

- The model designing step needs more description especially the role of CBCT and the correlation with the 3D scanning.

 

**Fig 3:**

- Just mention Bar chart or column chart.

 

**Fig 4:**

- Indicate which mode of failure that figure is.

 

**Discussion:**

- Paragraphs 2 to 6 are not necessary. Go straight for the interpretation after paragraph 1.
- Reference 33 has no journal. 

Is the work clearly and accurately presented and does it cite the current literature?

Yes

If applicable, is the statistical analysis and its interpretation appropriate?

Partly

Are all the source data underlying the results available to ensure full reproducibility?

Yes

Is the study design appropriate and is the work technically sound?

Yes

Are the conclusions drawn adequately supported by the results?

Yes

Are sufficient details of methods and analysis provided to allow replication by others?

Yes

Reviewer Expertise:

Prosthodontics, Esthetic dentistry, Digital dentistry, Implantology

I confirm that I have read this submission and believe that I have an appropriate level of expertise to confirm that it is of an acceptable scientific standard.

# Response

**Published:** 2021-06-01

**Authors:** Omnia Nabil

**Affiliations:** Cairo University, Egypt

Thank you very much for your careful reading, useful comments and accepting our article.

 Your effort and time are much appreciated.

 A second version of the article has been submitted, which has the following changes made in response:

**Materials and Methods:**

- *Why didn’t you consider doing mechanical cyclic loading before the fracture resistance testing? I think it would have been more clinically significant.*

In our study we focused on the impact of the occlusal preparation design only on the fracture resistance and stress distribution among a ceramic crown-molar structure. A static load fracture test was preferred to avoid the addition of any variables that can be caused by fatigue testing.

  

- *More details concerning teeth dimensions. *

The bucco-lingual dimension of crown as measured between the buccal and lingual maximum convexities was (10.5 ± 0.25 mm), while the mesio-distal dimension at cervix was (9 ± 0.25 mm) as measured using a digital caliber (Harbor Freight Tools, CA, USA).

  

- *Access cavity design and dimensions should be also described as all these details are important in behavior of the teeth during fracture resistance testing. And also, please mention the master apical file size.*

In all teeth, the access cavity was prepared with a round diamond bur which was directed at center. The undercuts of dentin had been removed with long shafted round bur and finally finishing and flaring was carried out by safe ended diamond bur to allow straight-line access for instrumentation of the apical part of the canal. The access cavity was triangular in case of single distal canal and trapezoidal in case of 2 distal canals, with the lesser base corresponding to the distal wall.

 Rotary root canal preparations were then performed with a series of ProTaper Ni-Ti rotary instruments (Dentsply Maillefer, Switzerland). The canals were prepared to F2 (D1 diameter 0.25 mm), while larger canals were shaped to F3 (D1 diameter 0.3 mm).

  

- *To which level were the teeth mounted in the epoxy resin?*

The mid-facial extent of the cementoenamel junction was located 2 mm coronal to the resin top surface.

  

- *There should be more elaboration on specifics of teeth preparation designs, especially occlusal designs. A diagram would be highly beneficial. The photograph is not that descriptive.*

A diagram has been added.

  

- *What were the details of crowns dimensions and standardization during designing?*

With the purpose of standardization, the restoration parameters were fixed for all the restorations with the radial spacer set at 60 µm, minimal thickness occlusal 1500 µm and minimal thickness radial 1200 µm. The restoration’s position in 3D (buccolingually, mesiodistally and occlusocervically) was adjusted by rotation tools to follow the anatomy of the prepared tooth. The distance between central groove of restoration and the occlusal surface of tooth was standardized at 1.5 mm in all restorations.

**Testing:**

- *Indicate the points of load application during fracture resistance testing?*

3 Loading points: 2 points on the inner inclines of the mesiobuccal and distobuccal cusps and 1 point on the inner incline of the mesiolingual cusp.

 A figure has been added

**Statistical analysis:**

- *There are no details. What type of tests or analysis did you perform?*

Data analysis was performed using IBM SPSS, version 21 (SPSS, Chicago, IL, USA). Student t-test was used to detect significance between two groups which was set at 5% for all statistical analyses and confidence interval at 95% such that  P values ≤0.05 were considered to be statistically significant

**FEA:**

- *The model designing step needs more description especially the role of CBCT and the correlation with the 3D scanning.*

CBCT 3D scanning of two of the prepared samples (one for each group) and their corresponding crowns was done before cementation.

 After scanning, data were exported in DICOM format. Mimics software version 17 (Materialise, Belgium) was used for segmenting the scanned objects into separate elements.

 A definitive threshold level was set to show each element of the scanned samples most clearly with minimal interference from the surrounding structures, and once segmentation was completed the software automatically calculated the element’s volume.

 The resulting STL files were opened separately on Meshmixer software version 3.3.15 (Autodesk Inc., USA) for the improvement of mesh quality and its refinement. Now we have the components of crown-tooth structure as separate elements of known volumes that can be assembled together in STL file format of improved quality.

 A figure has been added to show components of STL files as separate elements

**Fig 3: Just mention Bar chart or column chart.**

- Column chart.

 

**Fig 4: Indicate which mode of failure that figure is.**

- Mode of failure III

 

**Discussion:**

- *Paragraphs 2 to 6 are not necessary. Go straight for the interpretation after paragraph 1.*

We think that justifying the important steps in the methodology would be beneficial for other readers to justify our choices.

   

**References:** *Reference 33 has no journal.*

 It is a reference to our article account on Open Science Framework to access all the extended data of our work.

# Peer review: Reviewer response for version 1

**DOI:** 10.5256/f1000research.21329.r57384

**Published:** 2019-12-05

**Authors:** Gürel Pekkan

**Affiliations:** 1 Department of Prosthodontics, Faculty of Dentistry, Tekirdag Namik Kemal University, Tekirdağ, Turkey

The manuscript is well organised and well written. However, it needs more literature review in the introduction as well as discussion of some points that are listed below:

1. In the introduction, give more literature review about the core build-up materials and techniques.
2. Give more info about the prosthetic materials and the reasons for the choice of different restorative materials in endodontically treated teeth.
3. Please discuss the ball-contact points and localization in the test configuration that would affect the force distribution in the tooth.

Is the work clearly and accurately presented and does it cite the current literature?

Yes

If applicable, is the statistical analysis and its interpretation appropriate?

Yes

Are all the source data underlying the results available to ensure full reproducibility?

Yes

Is the study design appropriate and is the work technically sound?

Yes

Are the conclusions drawn adequately supported by the results?

Yes

Are sufficient details of methods and analysis provided to allow replication by others?

Yes

Reviewer Expertise:

NA

I confirm that I have read this submission and believe that I have an appropriate level of expertise to confirm that it is of an acceptable scientific standard, however I have significant reservations, as outlined above.

# Response

**Published:** 2021-06-01

**Authors:** Omnia Nabil

**Affiliations:** Cairo University, Egypt

Thank you very much for your careful reading and useful comments.

 Your effort and time are much appreciated.

 A second version of the article has been submitted, which has the following changes made in response:

1. In the introduction, more literature review has been given about the core build-up materials and techniques. (Paragraphs 1 and 2)
2. In the introduction, more info about the prosthetic materials and the reasons for the choice of different restorative materials in endodontically treated teeth has been added. (Paragraphs 3, 4 and 5)
3. In the discussion, the ball-contact points and direction of load have been addressed. (Paragraph 4)
